# The Role of Autophagy in White Adipose Tissue Function: Implications for Metabolic Health

**DOI:** 10.3390/metabo10050179

**Published:** 2020-04-30

**Authors:** Mercedes Clemente-Postigo, Alberto Tinahones, Rajaa El Bekay, María M. Malagón, Francisco J. Tinahones

**Affiliations:** 1 Department of Cell Biology, Physiology and Immunology, Instituto Maimónides de Investigación Biomédica de Córdoba (IMIBIC)-Reina Sofia University Hospital, University of Cordoba, Edificio IMIBIC, Av. Menéndez Pidal s/n, 14004 Córdoba, Spain; bc1mapom@uco.es; 2 Unidad de Gestión Clínica de Endocrinología y Nutrición (Hospital Universitario Virgen de la Victoria), Instituto de Investigación Biomédica de Málaga (IBIMA), Universidad de Málaga, Campus Teatinos s/n, 29010 Málaga, Spain; albertotruano@gmail.com; 3 Unidad de Gestión Clínica de Endocrinología y Nutrición (Hospital Universitario Regional de Málaga), Instituto de Investigación Biomédica de Málaga (IBIMA), Universidad de Málaga, Campus Teatinos s/n, 29010 Málaga, Spain; elbekay@gmail.com; 4 Centro de Investigación Biomédica en Red (CIBER) Fisiopatología de la Obesidad y Nutrición (CIBEROBN), Instituto de Salud Carlos III (ISCIII), 28029 Madrid, Spain

**Keywords:** adipose tissue, adipocyte, autophagy, obesity, diabetes, metabolism

## Abstract

White adipose tissue (WAT) is a highly adaptive endocrine organ that continuously remodels in response to nutritional cues. WAT expands to store excess energy by increasing adipocyte number and/or size. Failure in WAT expansion has serious consequences on metabolic health resulting in altered lipid, glucose, and inflammatory profiles. Besides an impaired adipogenesis, fibrosis and low-grade inflammation also characterize dysfunctional WAT. Nevertheless, the precise mechanisms leading to impaired WAT expansibility are yet unresolved. Autophagy is a conserved and essential process for cellular homeostasis, which constitutively allows the recycling of damaged or long-lived proteins and organelles, but is also highly induced under stress conditions to provide nutrients and remove pathogens. By modulating protein and organelle content, autophagy is also essential for cell remodeling, maintenance, and survival. In this line, autophagy has been involved in many processes affected during WAT maladaptation, including adipogenesis, adipocyte, and macrophage function, inflammatory response, and fibrosis. WAT autophagy dysregulation is related to obesity and diabetes. However, it remains unclear whether WAT autophagy alteration in obese and diabetic patients are the cause or the consequence of WAT malfunction. In this review, current data regarding these issues are discussed, focusing on evidence from human studies.

## 1. Introduction

Obesity is considered a major health problem which prevalence has globally increased over the last decades [1,2]. Obesity, which is defined by an increased white adipose tissue (WAT) mass, entails an increased risk of several chronic illnesses including type 2 diabetes (T2D) and insulin resistance, cardiovascular diseases (CVD), hepatic steatosis and even several types of cancer [1,2,3]. Adipose tissue (AT) dysfunction, beyond AT mass, has been proposed as a key player in the development of obesity-related complications [4,5]. 

AT function is essential for the correct metabolic regulation [6,7]. There are distinct AT depots with divergent functions. WAT mainly stores excess energy as triglycerides after feeding and mobilizes lipids during fasting to meet the energy demands from other tissues. By contrast, brown (BAT) and beige or brite (brown-into-white) AT are thermogenic tissues that burn energy. Notably, WAT, BAT, and beige AT exert relevant functions as endocrine organs by releasing adipokines (or batokines in the case of BAT) such as hormones, cytokines, or microRNAs, which regulate and participate in the crosstalk with other metabolic organs (e.g., liver, muscle, brain). This allows the integration of metabolic signals to comply with body energy requirements depending on environmental cues [8,9,10]. In addition, different roles in energy homeostasis have been attributed to WAT depots depending on their location, i.e., visceral (vWAT) or subcutaneous (sWAT) [4]. Therefore, the maintenance of proper AT function is crucial in keeping metabolic health. 

Among the molecular and cellular processes involved in AT functioning, autophagy has emerged as a crucial cellular phenomenon for AT homeostasis [11,12]. Autophagy is a highly conserved homeostatic process essential for the survival of eukaryotic cells [13,14]. Damaged, redundant, or long-lived intracellular components are degraded into lysosomes through autophagy. Moreover, intracellular pathogens (and more recently demonstrated, extracellular microbial) and their components can be also eliminated by autophagy [15,16,17]. Besides allowing the turnover of organelles and intracellular molecules, autophagy is a source of macromolecules and nutrients during starvation. Autophagy is highly responsive to the nutritional status and is tightly regulated by the same mechanisms involved in the maintenance of metabolic homeostasis and the response to the feeding state e.g., insulin. Reciprocally, autophagy regulates many processes related to metabolism e.g., food intake by hypothalamic neurons, the differentiation and the fate of adipocytes or maintenance of β-cells, and hepatocytes structure and function for proper metabolic regulation [11,18,19,20]. However, there are many gaps regarding the precise relationship between autophagy and metabolic homeostasis. In the context of obesity and obesity-related diseases, the role of autophagy imbalance in AT remains unclear and somewhat contradictory, but their pivotal role in adipocyte fate and its close relationship with metabolism, highlight autophagy as an appealing target for targeting WAT dysfunction.

In this review, we summarize the latest findings regarding the role of autophagy in the physiology of WAT (the main contributor for the obese phenotype and related-metabolic disturbances) and the impact of WAT autophagy dysregulation on systemic metabolic homeostasis. We will particularly focus on how autophagy is involved in WAT expansion, which not only depends on the formation of new adipocytes and adipocyte growth but also on essential processes which define the limits of WAT expandability such as inflammation and fibrosis. Eventually, a comprehensive description and discussion of the current literature on human WAT autophagy regarding obesity and glycemic status is presented, including intervention studies for weight loss and metabolic improvement. We also highlight the current limitations and unsolved questions that need to be addressed in subsequent studies analyzing autophagy in WAT.

## 2. The Role of WAT Dysfunction in Metabolic Homeostasis

WAT is a key player in metabolic homeostasis not only as an energy reservoir but also as an endocrine organ [7]. Adipocytes store excess energy in the form of triglycerides inside intracellular lipid droplets during feeding, and releases free fatty acids into the circulation to meet body energy requirements during fasting [9]. These processes have to be tightly regulated as the lack of excess energy storage and/or enhanced fatty acid release can have serious consequences for the health of the individual. In addition, WAT participates in metabolic endocrine regulation by secreting endocrine mediators which not only play a relevant role in integrating metabolic signals [8,9,21], but also in other biological processes such as reproduction or immune response [22,23]. Furthermore, adipocytes with thermogenic capacity in WAT, i.e., beige adipocytes, which emerge in response to environmental cold and other stimuli (*browning*), also participate in the maintenance of WAT and body homeostasis [9,10,24]. Interestingly, brown and beige adipocytes can also transdifferentiate into white adipocytes (*whitening*) [25,26].

To house the energy surplus, adult WAT expansion occurs by hypertrophy (adipocyte size increase due to triglyceride accumulation, i.e., lipogenesis) and/or by hyperplasia (increase in cell number by the proliferation of adipocyte precursors, preadipocytes, and their differentiation into new adipocytes, i.e., adipogenesis). The formation of new vasculature (angiogenesis) for correct oxygen and nutrient supply occurs concomitantly [27]. Together with these phenomena, extracellular matrix (ECM) remodels to accommodate these cellular changes [4,9,28].

The increased WAT size in obesity has been considered a major contributor for the development of obesity comorbidities [27,29]. However, there are also obese subjects who are apparently metabolically healthy without alterations related to metabolic syndrome (high glucose and triglyceride levels, high blood pressure, low HDL-cholesterol levels, insulin resistance) [30,31,32,33]. On the other hand, some non-obese individuals display an unfavorable metabolic profile and are at high risk of CVD [34,35]. What is more, patients with lipodystrophy (i.e., abnormal WAT distribution characterized by total or partial loss of this tissue) have the same metabolic alterations as obese subjects [36,37]. Given these paradoxical phenotypes regarding adiposity and metabolic dysregulation, it has been proposed that it is not the total body fat mass but the capacity of WAT to healthily expand which determines the metabolic status of the individual [5,38]. However, the precise mechanisms underlying the pathogenic processes which determine the threshold for the transformation of WAT into a dysfunctional organ, are not fully understood.

It has been suggested that continuous energy flow to WAT can result in tissue stress and failure. Distressed hypertrophic adipocytes display an impaired lipogenic capacity and an altered proinflammatory secretory profile. This proinflammatory state further impairs WAT function by interfering with local insulin signaling (which governs transcriptional regulation of nutrient up-take and lipid storage by adipocytes and adipocyte formation), recruiting pro-inflammatory immune cells and promoting tissue fibrosis (ECM maladaptive remodeling). WAT fibrosis develops due to an altered synthesis and degradation of the ECM by tissue-resident cells, and promotes further adipocyte dysfunction by mechanical stress (due to increased contact with neighboring cells and the ECM), limiting cell size increase [39,40,41]. Therefore, a harmful vicious circle is initiated in dysfunctional WAT. This pathological response of WAT is usually related to hypertrophic adipocytes rather than hyperplasic ones. Large hypertrophic adipocytes are associated with an impaired glucose metabolism and metabolic alterations while small adipocytes are often correlated with decreased susceptibility to developing diabetes [4,7]. As they increase in size, adipocytes experience mechanical and hypoxic stress. At the intracellular level, hypertrophic adipocytes exhibit endoplasmic reticulum (ER) stress, mitochondrial dysfunction, and oxidative stress, which come together with the activation of inflammatory pathways [9,42]. By contrast, hyperplasic adipocytes and adipogenesis are associated with reduced hypoxic stress and inflammation, providing a lipid sink that can alleviate, at least in part, the inability of hypertrophied, dysfunctional adipocytes to store lipids [43]. 

Inefficient lipid sequestration by adipocytes causes high plasma lipid levels (hyperlipidemia) and toxic lipid accumulation (lipotoxicity) in non-fatty organs such as the liver, muscle, or heart, which has been linked to the development of systemic insulin resistance [9,21,44]. Additionally, secreted proinflammatory factors from WAT into the circulation, also compromise insulin signaling and functioning of other organs [21,28,45]. Notably, the type of WAT depot, visceral (vWAT) or subcutaneous (sWAT), also have differential roles in energy dysregulation during metabolic disturbance [4]. vWAT accumulation (central obesity) is typically associated with metabolic complications, while increased sWAT (peripheral obesity) has been related to a more favorable metabolic profile. The opposite relationship of increased vWAT and sWAT with metabolic health may be due to the intrinsic characteristics and differential functions of each WAT depot [9,21].

Although food intake overload was initially proposed as the triggering factor for WAT dysfunction, it is not fully clear what comes first: adipocyte dysfunction, inflammation, or fibrosis. Therefore, the study of cellular and molecular mechanisms involved in these processes is of equal relevance in order to understand WAT pathophysiology and its consequences in body metabolic health. Within this context, autophagy has been positioned at the crossroad of several essential cellular processes for WAT homeostasis. Roles reported for autophagy in the different processes involved in WAT expansion are discussed in the following sections. 

## 3. Autophagy

Autophagy is a catabolic process that eliminates redundant or damaged intracellular components by delivering them to lysosomes. This degradative process is essential for maintaining cell homeostasis (constitutive autophagy), but can be also highly induced (adaptive autophagy) during starvation (to provide nutrients for energy supply and macromolecules), stress conditions (e.g., oxidative o ER stress, which result in an increase of damaged molecules), or infection (for intracellular or extracellular (secretory autophagy) pathogen degradation [13,17,46,47,48]. Consequently, autophagy is essential for the correct functioning of every organ in the body. Concordant to the role of autophagy as an adaptive response to nutrient deprivation, this process is mainly regulated by mediators involved in nutrient sensing (e.g., insulin, mTOR, glycogen) and is intimately related to metabolic homeostasis [13,46,49,50,51,52,53]. Furthermore, autophagy up-regulation is associated with inflammatory response and cellular stress related to adipocyte dysfunction [54,55].

Autophagy activation has been mainly related to cell survival. In fact, upregulated basal autophagy can promote longevity and prevent premature aging [56]. However, an enhanced autophagic flux may also lead to cellular death due to excess cytoplasm degradation [57]. Notably, autophagy deficiency also has detrimental effects on cell functioning by the accumulation of dysfunctional proteins and organelles and/or diminished defenses against pathogens, which can also lead to cell death [55,58]. The relevance of tight regulation of autophagy is highlighted by the association between autophagic imbalance and several disorders including diabetes, obesity, cancer, or neurodegeneration [54,59,60]. Within this context, dysregulated autophagy can be related to the development of the WAT and systemic low-grade inflammatory state present in metabolic diseases [61]. 

Mammalian autophagy has been extensively reviewed elsewhere [13,62,63]. Briefly, according to the delivery route to lysosomes, there are three types of autophagy: (1) macroautophagy, which implies the sequestering of part of the cytoplasm and intracellular components into a double-membrane vesicle, so-called autophagosome, which fuses with the lysosome; (2) microautophagy by which lysosomal membrane invaginations directly engulf cytoplasmic material; and (3) chaperone-mediated autophagy (CMA) which does not require the sequestration of cargo (targets for degradation) by biological membranes. In CMA, cargo is selectively delivered via chaperones to lysosomes. The cargo-chaperone complex is recognized and translocated into lysosomes through a lysosomal membrane translocation complex composed of lysosome-associated membrane protein type (LAMP)-2 [47]. It was initially thought that, in contrast to CMA, macro- and microautophagy were non-selective ways of autophagy, mainly responsible for adaptive bulk autophagy in response to nutrient deprivation and other stressful conditions. To date, it is well established that these two vesicle-mediated ways of autophagy can also selectively degrade molecules and organelles [47,48,64,65,66]. 

### Macroautophagy Pathway

Macroautophagy (hereafter referred to as autophagy) is the most studied type of autophagy. Although non-selective bulk autophagy was mainly described to be up-regulated as an adaptive response, it also occurs at low basal levels to facilitate the turn-over of cell components and recycling. Additionally, selective autophagy removes specific dysfunctional and/or redundant organelles and molecules as well as intracellular pathogens by means of receptor-mediated cargo recognition [48,64]. Selective autophagy also provides specific nutrients depending on environmental cues, allowing the adaptation to lipid imbalance or amino acid, iron, or glucose shortage [13,48]. Autophagic processes are also classified according to the cargo to be degraded, e.g., mitochondria (mitophagy), aggregated proteins (aggrephagy), peroxisomes (pexophagy), lipid droplets (lipophagy), intracellular pathogens (xenophagy), and ER (reticulophagy) among others [48]. Interestingly, selective autophagy plays a key role in cellular remodeling which becomes especially relevant in WAT adaptation to nutritional status [11,48], as discussed later. 

The autophagy process is orchestrated by core machinery consisting of several “autophagy-related genes” (ATG) [67]. The participation of the different ATGs occur in consecutive phases that can be summarized as: (1) initiation or induction, (2) nucleation, (3) elongation and autophagosome formation, (4) autophagosome maturation and (5) autophagosome-lysosome fusion and cargo degradation. 

Autophagy induction leads to the recruitment of ATGs to the phagophore assembly site (PAS) and nucleation of the phagophore (a cup-shaped doubled-membrane which is the initial piece of the autophagosome). This process is initiated by de-phosphorylation, dissociation from mTORC1, and consequent activation of the serine/threonine kinase ULK1/2 which forms part of the ULK1/2 complex together with ATG13, ATG101, and FIP200. ULK1/2 complex triggers phagophore nucleation by phosphorylating and activating several components of the PI3KC3 complex (VPS34-Beclin1-Ambra1-ATG14-p115) [46,49,50]. PI3KC3 complex enhances local PI3P production at PAS and recruits the effector proteins WIPIs and DFCP1 to the omegasome (ER-emanating membrane domain) which initiates double-membrane vesicle nucleation [68,69,70]. ATG12-ATG5-ATG16L complex (previously conjugated by the action of ATG7 and ATG10) is recruited by WIPI by binding ATGL16. 

A second conjugation system that involves ATG8-family proteins (e.g., LC3 and GABARAPs) is required for phagophore elongation. LC3 precursor is processed by ATG4, generating the soluble LC3 form (LC3-I). LC3-I is conjugated to membrane phosphatidylethanolamine (PE) by the action of ATG7, ATG3, and the ATG12-ATG5-ATG16L complex. This gives rise to the membrane-associated lipidated LC3 form (LC3-II) [71]. Lipidated ATG8 family members attract components that contain the LC3-interacting region (LIR) for phagophore elongation but also serve as cargo recruiting agents in selective autophagy by interacting with LIR of cargo receptors e.g., p62 or sequestosome. These cargo receptors recognize the cargo through degradation signals such as ubiquitin. 

Gradual elongation of the phagophore curved membrane results in a sphere which rounds part of the cytoplasm, including autophagic cargo and eventually seals, giving rise to the doubled-membrane autophagosome which encapsulates and isolates cytosolic material together with the cargo. Though PAS is thought to be mainly located at ER-emanating membrane domains (named omegasomes), other organelles such as the plasma membrane or the Golgi complex have been recently proposed as PASs [72]. It is thought that a mechanism mediated by ATG9-containing vesicles contributes to phagophore expansion through the addition of these cellular membranes [73]. 

Once autophagosomal membranes seal, autophagosome maturation takes place involving ATGs clearance from the outer membrane and the recruitment of machinery for lysosomal delivery and fusion [74,75]. After dissociation of autophagy protein machinery, the exterior membrane of the autophagosome fuses with the lysosomal membrane, resulting in the release of a single-membrane autophagic body into the autolysosome that, together with the autophagic cargo, will be degraded by lysosomal hydrolytic enzymes and the resulting usable nutrients are released back to the cytoplasm [13,63].

Autophagy is tightly regulated by nutrient sensors such as mTORC1 and AMPK. During the fed state, insulin activates mTORC1 which binds and phosphorylates ULK1/2 and consequently, avoids autophagy initiation. By contrast, under fasting conditions, mTORC1 is inactivated by AMPK triggering autophagy induction by releasing ULK1/2 from mTORC1 [13]. 

Even if the most studied participants in autophagy have been referred to in this review, other mediators are known to be involved in this process and the detailed autophagy pathway has been reviewed elsewhere [63,71]. 

## 4. Autophagy Involvement in WAT Expandability: Clues from Experimental Data

### 4.1. Autophagy and White Adipogenesis

**Figure 1:**
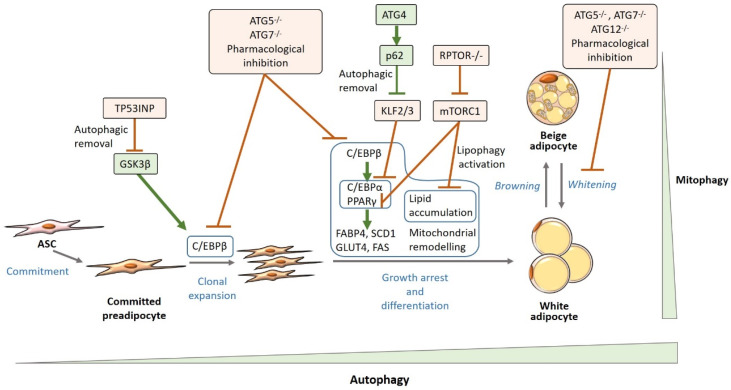
Autophagy modulates white adipogenesis. Autophagy is induced during adipogenesis. Genetic and pharmacological inhibition of autophagy has been shown to repress early and late adipogenesis. Similarly, mitophagy is required for whitening of beige adipocytes. In fact, autophagy inhibition maintains beige phenotype. Despite most of the studies suggest that autophagy promotes adipogenesis and the white adipocyte phenotype, others have found opposite evidence, e.g., autophagic removal of adipogenic activators such as GSK3β or lipophagy activation by mTORC1 inhibition, which results in impaired lipid accumulation in terminal phases of adipogenesis together with PPARγ downregulation. Here we present an overview of the evidence relating autophagy with white adipogenesis. Reddish elements depict negative effects on adipogenesis, while green elements favor white adipocyte formation.

Apart from the well-described role of autophagy as a nutrient provider during energy deprivation, autophagy is an essential process for cell remodeling and differentiation [76,77]. In this vein, adipocyte differentiation is highly dependent on autophagy (Figure 1) [76,77,78,79,80,81,82,83]. 10-years ago, several authors reported the relationship between autophagy deficiency (i.e., deletion of specific *Atg* genes) and an impairment in white adipogenesis in cellular and animal models [78,79,80]. Adipocyte-specific (aP2+ cells) *Atg7* knock-out mice showed reduced WAT mass irrespective of diet (control vs. high-fat diet) while increasing body insulin sensitivity [78,80]. Similarly, pharmacological inhibition of autophagy also led to resistance to high-fat diet-induced obesity and insulin resistance [83]. This highlights the relevance that WAT autophagy can have in systemic metabolic regulation and the study on the precise mechanisms which link WAT autophagy and white adipogenesis has attracted great attention. 

Adipogenesis is a biphasic process by which multipotent, fibroblast-like, adipose mesenchymal stem cells (ASCs) are transformed into mature unilocular adipocytes. Mature adipocytes mainly consist of an enormous lipid droplet which occupies most of the cytoplasm with the consequent removal of a great part of the organelles. Consequently, the cytoplasm of ASCs has to undergo profound remodeling. Firstly, fibroblast-like progenitor cells enter the commitment pathway and, without morphological modifications, restrict their fate to the adipocyte lineage by giving rise to committed preadipocytes. Secondly, preadipocytes differentiate into mature adipocytes [7,27]. During the early steps of differentiation, precursor cells undergo a proliferation phase namely clonal expansion. In this step, the transcription factor CCAAT-enhancer-binding protein (C/EBP)-β seems to be activated and is required for the regulation of mitotic clonal expansion [84,85]. Afterward, C/EBPβ is phosphorylated by glycogen synthase kinase-3β (GSK3β) driving the adipogenic process forward [86]. C/EBPβ binds to C/EBP regulatory elements in the promoter region of key adipogenic transcription factors, i.e., peroxisome proliferator-activated receptor (PPAR)-γ and C/EBPα. These two transcription factors induce and maintain the expression of key adipogenic genes such as fatty acid-binding protein (FABP)-4, GLUT4, or adiponectin, favoring lipid accumulation into the lipid droplet and insulin responsiveness in mature adipocytes [27,87,88]. 

As reported by our group and others, the proliferation and differentiation capacities of ASCs are compromised in obesity [89,90,91,92,93]. The impairment in ASCs biology is more pronounced in those patients with metabolic alterations and abdominal obesity, with differences between fat depots [92,93,94]. However, the precise mechanisms underlying these metabolic and obesity-induced changes in ASCs are not completely elucidated. Interestingly, it has been described that autophagy is involved and gradually induced during adipocyte differentiation (i.e., increasing number of autophagosomes in the cytoplasm, higher expression of autophagic markers, e.g., LC3-II, coupled with a suggested increase in autophagic flux), which places autophagy as a putative key element for white adipogenesis [79,83,95]. Notably, although mature adipocytes were thought to be terminally differentiated cells that just undergo changes in their size, recent evidence suggests that they can also de-differentiate into fibroblast-like preadipocytes [7,9]. In addition, beige adipocytes, which have been proposed to emerge by either differentiation from specific precursors or transdifferentiation from white mature adipocytes, can also revert to a white-like adipocyte phenotype (*whitening*) [9,22]. While the role of autophagy in adipocyte dedifferentiation is yet to be determined, evidence shows that the *whitening* program can also be highly regulated by autophagy (Figure 1) [26,96,97].

Baerga et al., showed that autophagy deficiency by *Atg5* deletion in mouse embryonic fibroblasts (MEF) induced for adipogenic differentiation led to an impairment in adipogenesis, specifically in the later steps related to morphological modifications, and eventually to cell death. Similar results were obtained when wild-type MEFs were treated with the autophagy inhibitor chloroquine [79]. In this line, further studies confirmed that autophagy inhibition in 3T3-L1 cells (the most used model for adipogenesis [98]) by *Atg5* or *Atg7* silencing, led to an impairment in triglyceride accumulation during adipogenesis, likely as a consequence of the observed reduced expression of adipogenic factors (C/EBPα and PPARγ and their targets FABP4, fatty acid synthase (FAS) and stearoyl–coenzyme A desaturase (SCD)-1). Similar results were seen when wild-type 3T3-L1 cells were exposed to pharmacological inhibition (3-methyladenine, ammonium chloride, and leupeptin) of autophagy [80,83]. In light of these findings, it was initially thought that autophagy was solely involved in late preadipocyte differentiation by enhanced lipid accumulation and favoring the last stage of adipogenesis towards a mature adipocyte. However, the study carried out by Singh et al., also revealed that C/EBPβ (involved in preadipocyte clonal expansion and the activation of transcription factors required for adipocyte maturation) was downregulated in both *Atg5* and *Atg7* deficient 3T3-L1 cells [80]. The effect of autophagy on early adipocyte differentiation was corroborated by Skop et al., who found that autophagy inhibition downregulated preadipocyte mitotic clonal expansion, and impaired mitochondrial remodeling in 3T3-L1 preadipocytes [95].

Even though specific deletion of *Atg5* or *Atg7*, which is supposed to impair autophagy, has been related to beneficial metabolic effects, studies on the deletion of other various autophagic genes reported opposite effects. Adipocyte-specific *p62*-deficient mice developed obesity irrespective of diet (control diet or high-fat diet), displayed impaired glucose tolerance, and diminished insulin sensitivity. In addition, contrary to *Atg7*-deficient mice, WAT from adipocyte-specific *p62*-deficient mice had greatly enlarged hypertrophic adipocytes as well as higher macrophage infiltration and expression of inflammatory markers. Curiously, no changes were reported regarding the expression of adipogenic, lipogenic or lipolytic markers. Moreover, there were no signs of enhanced free fatty acid mobilization. However, an impaired mitochondrial function on BAT, likely due to decreased quality of mitochondria was reported. This was related to an impaired BAT thermogenesis [99]. This remarks the importance of the crosstalk between different AT depots in regulating metabolic homeostasis. Another in vitro study on *Atg16l* deletion showed in the same vein, an association with impaired insulin responsiveness in MEFs. Impaired insulin sensitivity was due to decreased IRS1 protein content as a result of enhanced proteasome degradation [100]. Furthermore, mice with *Bif1* (a positive regulator of autophagosome formation) deficiency were more prone to develop obesity and hyperinsulinemia with aging or a high-fat diet. *Bif1* deficiency also promoted WAT expansion and the presence of hypertrophied adipocytes with decreased lipolytic rate and expression of ATGs [101]. 

These discordant findings according to the genetic model of autophagy deficiency give an idea surrounding the complexity of the autophagic pathway and its relationship with WAT physiology. Because of the pleiotropic role of autophagy regulating different cellular processes, autophagy deficiency may have different general consequences depending on the balance of autophagy-regulated pathways. In addition, non-canonical functions of autophagic genes have been reported [102], which could also account for the difference depending on the autophagic gene deleted. 

#### Mechanisms Underlying the Relationship Between Autophagy and White Adipocyte Biology

##### Autophagic Regulation of Adipogenic Factors

Although the effect that autophagy deficiency can have on adipogenesis has been reported, mechanisms by which autophagy alters WAT physiology are poorly understood. Some studies have shown that autophagy can remove specific inhibitory adipogenic factors [83,103]. It has been shown that the activation of *Atg4b* transcription (required for LC3-I formation) by C/EBPβ is required for the p62-mediated autophagic removal of krüppel-like factors 2 and 3 (KLF2/3), which are transcriptional repressors of *Cebpa* and *Pparg* [103]. Also, autophagy prevents proteasome-dependent PPARγ2 degradation as demonstrated by pharmacological and genetic models of autophagy deficiency [83]. These findings suggest a reciprocal regulation of autophagy and adipogenesis and also give mechanistic clues on how adipogenesis can be regulated by autophagy. However, not all studies go in the same direction. The role of autophagy in limiting the availability of adipogenic activators has also been reported. A recent study indicates that GSK3β is sequestrated into late endosomes via-autophagy with the resulting inhibition of adipogenesis in human subcutaneous preadipocytes [104]. 

##### Mitophagy

In addition to the degradation of adipogenic regulators and the consequent modulation of adipogenic transcription factors, it has been proposed that autophagy can directly modulate the morphological transformation of the fibroblast-like preadipocyte into a mature adipocyte by modulating organelle content [26]. Mitophagy, selective removal of mitochondria by autophagy, is a relevant player in mitochondrial content modulation as well as in mitochondrial quality control. Mitophagy is induced after mitochondrial depolarization. PTEN-induced putative kinase (PINK)-1 and Parkin mitophagy pathway is the most characterized mechanism of mitophagy [105]. Mitochondrial depolarization leads to the translocation of PINK1 to the outer mitochondrial membrane, where it is activated. PINK1 phosphorylates the cytosolic ubiquitin E3-ligase, Parkin. The accumulation of ubiquitin chain linkages on the mitochondrial outer membrane as a result of PINK1 and Parkin action, allows the binding of mitophagy receptors that recognize mitochondrial ubiquitin marks (e.g., p62, FUNDC1 or BNIP3). These receptors interact with mitochondria and LIR at the nascent autophagosome, so mitochondria is included into autophagosome for lysosomal degradation [105,106].

Mitophagy has been highlighted as an essential process for white adipogenesis. Evidence have shown that adipogenic differentiation is associated with an increase in the number of autophagosomes containing mitochondria [81]. Cummins et al., found that, after a high-fat diet, adipocytes underwent remarkable metabolic changes prior to WAT macrophage infiltration, including loss of mitochondrial biogenesis followed by the downregulation of mitochondrial proteins [107]. Besides its role in white adipogenesis, the modulation of mitochondrial content and quality is essential for the *browning* of WAT, as well as for the *whitening* of beige and brown adipocytes [76,108]. Within this context, Taylor et al., observed a downregulation of Parkin-mediated mitophagy during the *browning* of WAT [108]. By contrast, the reversion of beige adipocytes to the non-thermogenic, white adipocyte-like state, depends on autophagic mitochondrial removal as demonstrated by Altshuler-Keylin et al. *Atg5* or *Atg12* deletion or pharmacological inhibition of autophagy in animal models, promoted UCP1 retention and the maintenance of beige adipocyte characteristics. These effects were related to less susceptibility to develop diet-induced obesity (DIO) and insulin resistance [109]. In this line, both animal and in vitro models of *Atg7* deficiency, showed that *Atg7*-deficient WAT and cultured cells displayed atypical brown-like morphology with high mitochondrial content, multilocular aspect with smaller lipid droplets and increased cytoplasm compared to the typical unilocular aspect of white mature adipocytes with a single large lipid droplet, few mitochondria and scarce cytoplasm [78,80]. A recent study has indeed shown that autophagy inhibition by *Atg7* deficiency prevents glucocorticoid-induced adiposity by suppressing BAT *whitening* [96]. 

High-fat diet consumption is associated with the *whitening* of BAT together with enhanced expression of mitophagy markers [97]. An increased autophagic flux, Parkin and PINK1 expression, and the number of mitochondria closely located to autophagosomes, was reported after high-fat diet feeding, which suggests that response of WAT to a high-fat diet, at least in part, depends on mitochondrial remodeling [107]. Intriguingly, a recent study showed that mice lacking the mitophagy receptor *Fundc1* developed an impaired response to a high-fat diet with more severe obesity and insulin resistance, and more infiltration of proinflammatory macrophages [106]. This could be, at least in part, because mitophagy is also required for maintaining proper mitochondrial function and avoiding cell dysfunction [105]. Thus, beyond the relationship with brown/beige adipocyte functioning, changes that white adipocytes undergo in order to adapt to a high-fat diet consumption, are also closely related to mitochondrial homeostasis and especially to mitophagy. 

Recent evidence suggests that mitophagy can be involved in the activation of cellular senescence in adipocyte precursors cells as well as in mature adipocytes. WAT senescence is a feature of obesity-associated metabolic disturbances [110] and autophagy has been found to be increased in senescent cells [111]. The accumulation of senescent cells in WAT impedes tissue renewal [112]. sWAT progenitor cells from obese and/or diabetic subjects showed a diminished ability to differentiate into mature adipocytes, which was associated with the induction of the senescence activators p53 and p16 [113]. Then, senescent progenitor cells have an impaired differentiation capability. In this vein, activation of p53 results in defective adipogenesis in stromal vascular fraction (SVF), while p53 down-regulation in MEFs or 3T3-L1 cells enhanced adipocyte differentiation and increased mitochondrial content [114,115]. However, whether autophagy and more precisely, mitophagy, is involved in these phenomena remains unsolved. On the other hand, activation of p53 also results in senescent mature 3T3-L1 adipocytes [110,113], while *p53* genetic ablation in mature adipocytes favored the beiging of sWAT by a reduction in mitophagy and increased number of mitochondria [116]. Thus, autophagy may also be involved in regulating adipocyte senescence which alters WAT plasticity and adipogenesis, and has serious consequences for metabolic health [110,117]. However, to date, the evidence regarding this issue remains scarce and further studies should be performed to clarify it.

##### Lipophagy

Lipophagy is the selective degradation of lipid droplets. It has been extensively studied in hepatocytes but also occurs in other cell types. In comparison with mitophagy, selective receptors for lipophagy have not been found [48]. Although the role of lipophagy in white adipogenesis is not yet understood, a recent study has pointed out that mTORC1 inhibition led to autophagic sequestration and lysosomal degradation of lipid droplets as well as lipolysis upregulation in vivo and in primary adipocytes [118]. As detailed in Section 3, activated mTORC1 represses autophagy. Regulatory associated protein of MTOR complex (RPTOR)-1 is required for mTORC1 activation and autophagy inhibition. Adipose-specific deletion (adiponectin-positive cells) of *Rptor1*, and consequently inhibition of mTORC1, promoted adipose lipophagy and affected adipocyte morphology with reduced lipid droplet size. The authors also concluded that this effect occurred in terminal phases of adipogenesis rather than early stages of differentiation and was accompanied by the downregulation of the adipogenic markers PPARγ and perilipin (PLIN)-1 [118]. When the effects of *Atg7* deficiency on lipophagy were analyzed, a decrease in the number of lysosomes containing lipid droplets was found, however, with a downregulation of adipogenic markers. Interestingly, *Atg7- Rptor1* double knock-out mice recovered WAT development and upregulated thermogenic gene expression in BAT compared to *Rptor1* knock-out [118].

These results highlight the complexity of adipogenic regulation by autophagy machinery, suggesting that other mechanisms than the autophagy pathway may be participating. In fact, previous studies described that mTORC1 repression (by either genetic ablation or by rapamycin) led to adipogenesis inhibition [82,119,120,121], similarly to *Atg5* and *Atg7* deficiencies [78,79,80], and was associated with decreased weight and enhanced mitochondrial respiration [119]. The explanation proposed for these contradictory results was the pleiotropic role that mTORC1 has beyond autophagy regulation (e.g., stimulation of anabolic pathways including protein, lipid, and nucleotide synthesis which allows cell growth) [122]. However, it is yet necessary to confirm whether the effects of mTORC1 deletion on adipose biology are due to autophagy induction, another mTORC1-regulated pathway, or both [11]. Regardless, the study by Zhang et al., confirmed that mTORC1 deletion has a direct effect on adipose lipophagy [118]. Evidence from human studies showed that the lipid droplet marker perilipin, colocalized with autophagic marker LC3 in subcutaneous adipocytes and that the occurrence of lipid droplet-containing autophagosome was higher in adipocytes from diabetic patients concomitantly with an attenuated mTOR expression. In view of these results, the authors hypothesized that lipophagy could be taking part in adipocyte lipolysis [123].

Contradictory evidence regarding the way in which autophagy affects adipocyte biology depending on the autophagy-related molecule analyzed, bring to light the complexity of this regulatory interplay and suggest that each participant can drive adipocyte differentiation and function in opposite ways, likely by both dependent- and independent-autophagy pathways. It is worth mentioning that most studies have focused their attention on adipocyte development, but few have been aimed at studying the crosstalk between autophagic mediators and mature adipocyte functioning. A recent study analyzed the effect of autophagy ablation by specific adipose deletion of *Atg3* and *Atg16l* in mature adipocytes. By using Cre-loxP recombination in vivo [124], the authors induced *Atg3* or *Atg16l* depletion at 8-weeks of age. Contrary to adipose-specific congenital *Atg5* or *Atg7* genetic deletion, postnatal *Atg3* or *Atg16l* deletion provoked peripheral insulin resistance regardless of diet or fat mass [125]. All this evidence remarks that autophagy can also be dependent not only on cell or tissue type, but also on the stage of differentiation or the cellular functional state, which would difficult the use of autophagy modulation as a therapeutic option. 

### 4.2. Autophagy and Extracellular Matrix Regulation: Implications for WAT Remodeling

WAT expansion and remodeling to store excess energy or to mobilize lipids in the case of nutrient deprivation is not only dependent on adipocytes themselves, but also depends on the extracellular environment that surrounds adipocytes and its precursors, which determines the physical space to grow in size and number. ECM, a complex three-dimensional protein network, constitutes the microenvironment that physically supports tissue-resident cells and maintains tissue structure [126,127]. ECM is mainly composed of fibrillary proteins such as collagens or elastin, and glycoproteins such as proteoglycans, fibronectin, or laminin, which altogether represent the core ECM [126]. Besides the core ECM, there are also a variety of ECM-associated proteins embedded in the ECM that modulates its composition by promoting degradation or deposition of core ECM components or controlling the interaction between ECM components [126,127,128,129,130]. 

The composition and spatial organization of the ECM define its degree of flexibility, establishing physical limits for cells to proliferate, to modify their shape and/or to grow. Moreover, cells (including adipocytes) respond to mechanical cues by activating different signaling pathways to modulate their physiology [40,131]. ECM also modulates the availability of soluble molecules such as growth and angiogenic factors, chemokines, or enzymes [127]. Furthermore, most of the ECM components interact with cells via integrin, activating intracellular signaling cascades, and connecting ECM with the cytoskeleton, which results in modulation of cell activity and motility [126]. Then, ECM not only provides mechanical stimuli, but it also controls cell activity and phenotype by modulating signals for cell survival, proliferation, differentiation, and motility [126,132]. ECM is such a relevant component for healthy WAT remodeling, that fibrosis (abnormal deposition and organization of ECM components) has been defined, together with inflammation and hypoxia, as one of the hallmarks of dysfunctional WAT [28,133]. Notably, ECM stiffness, defined by the composition and organization of fibrillary ECM components, reciprocally depends on cell production of ECM-core components as well as ECM-associated and ECM-modifying factors [40,94,134,135]. Therefore, proper regulation of ECM synthesis and degradation pathways is essential for maintaining healthy WAT remodeling. Major ECM-producing cells in WAT include fibroblast, myofibroblast, adipocyte precursors, mature adipocytes, and macrophages [40]. 

There are a number of studies that have shown the involvement of autophagy in fibrotic processes of several tissues and organs such as the liver, kidney, heart, cartilage, or pancreas [136,137,138,139,140]. Several studies have given clues regarding the modulation of fibroblast biology and the differentiation into profibrogenic myofibroblast by autophagy [141]. The relationship between ECM and autophagy is bidirectional: autophagy can modulate the synthesis and degradation of ECM components by tissue-resident cells, but ECM can also modify the autophagic cell response [140,142,143,144,145,146]. However, this relationship between ECM and autophagy has been poorly explored in WAT. One of the latest studies in WAT autophagy has reported the relationship between the autophagic pathway and the fibrogenic response of adipose precursors to a high-fat diet. For this purpose, Pdgfra-CreErt2 transgenic mice were used for generating conditional deletion of *Atg7* in adipocyte progenitor cells [147]. PDGFRα positive cell population (referred to as fibro/adipogenic progenitors due to their dual potential toward either fibroblast, white adipocyte, or beige adipocyte differentiation [148,149,150,151]), has been shown to be central for WAT fibrosis. Knock-out mice showed a decrease in the WAT expression of ECM components as well as in the fibrosis score (determined by collagen deposition) in response to a high-fat diet compared to control mice. These changes were independent of fat mass, adipocyte cell number or the inflammatory response. In vitro analyses suggest that the attenuation of WAT fibrogenic response to a high-fat diet is mediated by an alteration in the expression of components of the TGF-BMP signaling pathway [147]. Furthermore, the authors found signs of WAT *beiging* in *Atg7* knock-out mice which inversely correlated with the expression of different collagen subtypes [147]. Decreased WAT fibrosis due to *Atg7* deficiency could partly explain the previous evidence of metabolic health in adipose-specific *Atg7* knock-out mice despite an impaired adipogenesis [78,80]. 

Results from this study suggest that autophagy deregulation might also be involved in WAT fibrosis. However, previous evidence has shown contradictory results depending on which autophagy molecule is deleted [80,99,101], therefore further studies should be performed in order to elucidate the precise mechanisms and autophagic mediators involved in the fibrogenic WAT response. In addition, the differential relationship between autophagic and ECM synthesis and degradation pathways depending on the WAT cell type analyzed should not be ruled out. 

### 4.3. Autophagy and WAT Inflammation

Autophagy has been closely related to innate and adaptive immunity, not only by eliminating pathogens, but also by modulating immune cell functions such as antigen presentation, development and maintenance of immune cells, and cytokine production and secretion in both immune and non-immune cells [152,153]. In this regard, autophagy functions as a protective mechanism against infections but also against the host’s excessive response by participating in immunological tolerance and the negative regulation of the immune response [154]. In agreement with the well-known function that autophagy has in degrading pathogens (xenophagy), activation of pattern recognition receptors (PPRs), such as toll-like receptors (TLRs) or nucleotide oligomerization domain (NOD)-like receptors (NLRs), which recognize and bind to microbial components (i.e., pathogen-associated molecular patterns (PAMPs)) or damage-associated molecular patterns (DAMPs) resulting from endogenous damage, trigger autophagy (and more specifically, xenophagy) by recruiting various ATGs proteins, as reviewed elsewhere [155,156]. Conversely, once triggered, autophagy negatively regulates inflammation by inhibiting cytokine secretion and production (e.g., IL1β, IFN1), by IL1β degradation in the autophagosome and by directly inhibiting the inflammasome. In addition, the inflammasome is indirectly down-regulated by autophagy due to diminished IL1β availability [155,156,157]. In this manner, autophagy restrains the inflammatory response that otherwise may cause serious complications, including septic shock, allergies, or metabolic disorders [155]. 

Although this is the general outline of the relationship between autophagy and immunity, there are some controversial research findings. For instance, it has also been reported that NLRs can inhibit autophagy by interacting with Beclin-1 [158] and that inflammatory stimuli (i.e., lipopolysaccharides, LPS) lead to supposed autophagy impairment in bone marrow-derived macrophages determined by the qualitative increase in p62 protein levels that were explained by the authors as protein accumulation due to diminished lysosomal p62 degradation [159]. Moreover, the regulation of autophagy by cytokines has been described, though stimulation or inhibition is dependent on the precise cytokine and the tissue of action [154,160]. Thus, there are still some unresolved issues regarding how the crosstalk between these two processes takes place. The implication of autophagy in immune response has not only been related to infectious diseases, but also to chronic inflammatory diseases such as metabolic disorders [154]. Within this context, the role of autophagy in the low-grade inflammatory state of WAT has drawn attention.

WAT malfunction is characterized by chronic low-grade inflammation with an increased infiltration of immune cells, especially macrophages. Under pathological conditions, WAT accumulates a higher number of apoptotic adipocytes which favors macrophage infiltration and inflammation. Moreover, as mentioned above, hypertrophic adipocytes display oxidative and ER stress, leading to the activation of inflammatory cascades and the release of proinflammatory cytokines that attract more immune cells [22,42,133,161]. Recruited monocytes polarize to a M1-like pro-inflammatory phenotype (“classically activated macrophages”) at the expense of diminishing M2-like anti-inflammatory macrophages (“alternatively activated macrophages”) which contribute to WAT homeostasis [135,162]. Inflammatory mediators disrupt insulin signaling, which impairs even more WAT function. What is more, WAT releases pro-inflammatory molecules into the circulation which promote systemic insulin resistance and impairs cardiovascular function [39,41]. In addition to WAT insulin resistance, inflammation is related to the fibrotic process. Fibrosis is part of the inflammatory response for injured tissue regeneration. However, it has to revert once the damage has been repaired. Otherwise, tissue dysfunction due to increased fibrosis would develop [133]. Macrophages modulate ECM composition and it has been suggested that M2-like macrophages participate in healthy ECM remodeling [163]. 

In this scene, macrophage autophagy, which has been implicated in macrophage polarization and function, may be playing a relevant role in WAT immunological homeostasis. Macrophage-specific *Atg7* deletion promoted the shift to the M1-like phenotype. When exposed to a high-fat diet, macrophage-specific *Atg7* knock-out mice showed higher WAT macrophage infiltration and proportion of M1-like proinflammatory phenotype compared to wild-type mice, as reflected by the increase in M1-like and reduction in M2-like macrophage markers. Though no significant differences in weight were found, macrophage-specific *Atg7* knock-out mice displayed impaired systemic insulin sensitivity. Autophagy blockage was also associated with elevated macrophage reactive oxygen species (ROS) and IL1β secretion in vitro [159]. Therefore, autophagy deficiency seems to promote the proinflammatory response of WAT macrophages. Studies on macrophage-specific *Atg5* knock-out mice revealed similarities in macrophage polarization towards a pro-inflammatory M1-like phenotype. However, contrary to *Atg7* macrophage deficiency, no effect was seen in WAT inflammation in *Atg5* or *Atg16l* macrophage-deficient mice [164,165]. This points out the differences between genetic models of autophagy deficiency and the putative disparate roles of ATGs in regulating other processes than autophagy according to tissue type. Notwithstanding, a recent study that focused on WAT macrophage autophagy reported that LC3 content was higher in WAT macrophages from genetically obese mice compared to lean mice. In addition, results from this study suggest that the WAT microenvironment is able to influence resident macrophages by enhancing autophagic flux. Unfortunately, the inflammatory profile of these macrophages were not determined [166]. Assuming that WAT from genetically obese mice is inflamed [167], these findings may be somewhat puzzling if it is considered that autophagy dampens the inflammatory response.

Metabolic endotoxemia, i.e., high circulating LPS levels due to increased translocation from the gut into the circulation, might be one of the pieces to solve this puzzle. Besides the well-accepted concept that WAT dysregulation contributes to systemic inflammation and metabolic disruption, it has also been proposed that external stimuli can trigger WAT inflammation. Apart from the detrimental effects of elevated nutrient consumption which can impair metabolic function (e.g., saturated fatty acids or excess glucose), nutritional stress can promote a low-grade systemic inflammatory state by enhancing the translocation of bacterial products, such as LPS, from the gut to the circulation [168]. Consumption of high-fat meals has been shown to especially enhance LPS translocation as these molecules bind to chylomicron during lipoprotein particle assembly in enterocytes [169]. Moreover, modulation of the gut microbiota composition by detrimental dietary compounds such as saturated fatty acids, leads to gut barrier dysfunction with increased gut permeability favoring more LPS translocation [170]. The increased levels of circulating LPS from gut microbiota related to nutritional stress and metabolic status has been named as “metabolic endotoxemia” [168]. It has been proposed that LPS could bind to TLRs in WAT cells and trigger adipocyte dysfunction and inflammation. In this vein, our group has recently demonstrated that metabolic endotoxemia is directly related to human adipose dysfunction and inflammation and that low LPS concentrations directly affect the function of human adipocytes [171].

Taking into consideration that TLRs activate autophagy, this could account for the increased autophagy in WAT during the obese state and metabolic disruption [172,173,174,175]. In keeping with this hypothesis, *Atg16l* deficiency was associated with exacerbated IL1β production upon LPS stimulation [157], suggesting that the inflammatory response in obesity could be even worse in the case that autophagy would not be up-regulated. In fact, ex vivo autophagy inhibition in human or mouse WAT explants from healthy lean subjects/animals led to an increase in gene and protein expression of proinflammatory cytokines [172].

Accordingly, the protective role of WAT autophagy in obesity against the inflammatory response, a parallel increase in autophagy and proinflammatory cytokines, and macrophage infiltration in obese subjects compared to normoglycemic lean individuals has been described [174]. Intriguingly, in vitro studies revealed that hypertrophic 3T3-L1 adipocytes had diminished autophagy but increased inflammation. This inverse relationship was confirmed by autophagy induction with rapamycin (inhibitor of mTOR), which reduced the expression of inflammatory cytokines as well as by autophagy inhibition with lysosomal blocking agents which enhanced the expression of inflammatory cytokines [176]. These findings highlight the reciprocal regulation between autophagy and inflammation in WAT. However, the precise mechanisms and molecular and cellular players implicated in the crosstalk between inflammation and autophagy and how this can influence WAT adaptation to nutritional stress remains to be investigated. 

## 5. WAT Autophagy Status in Obesity and Diabetes: Evidence from Animal and Human Studies

Most of the evidence regarding the systemic metabolic consequences of disrupted autophagy in WAT comes from animal studies. Animal models of adipose-specific deletion of autophagic genes have demonstrated that WAT dysregulation due to impaired autophagy has serious consequences in systemic metabolic regulation. On the one hand, *Atg5* or *Atg7* knock-out mice were resistant to diet-induced weight gain with reduced white adipogenesis, but improved insulin sensitivity and reduced leptin levels [78,80]. If considered that adipogenesis is the way of WAT to healthily expand (as described in Section 2), the positive metabolic characteristics of these mice seem to be contradictory at first sight. However, the metabolic effects of *Atg5* or *Atg7* deletion might be mediated, at least in part, by the increase in lipid β-oxidation and *browning* of WAT [78,80] as well as by decreased fibrosis [147], which would explain the healthy metabolic profile despite decreased WAT adipogenesis. Another issue to be mindful of is that other studies on autophagy deficiency that targeted different autophagic genes (see Section 4.1), found opposite results with detrimental effects on adiposity and metabolism [99,100,101]. Despite the valuable knowledge obtained from animal models of adipose-specific autophagy deficiency, it remains unclear how the global autophagic process is modulated in obesity and metabolic diseases. 

Direct exploration of WAT autophagic status in relation to metabolic diseases is required for elucidating the actual mechanisms underlying the development of WAT malfunction related to obesity and metabolic dysregulation. In this vein, high-fat diet-induced obese mice had impaired autophagy in WAT [176,177,178]. More specifically, increased LC3-II and p62 protein levels in vWAT in DIO mice were reported, while ATG5 or Beclin-1 levels remained unchanged. Determination of autophagic flux in vWAT explants from DIO mice suggested an enhanced formation of autophagosomes, but impaired lysosomal function in obese vWAT [177]. Another study reported total autophagic suppression in high-fat diet-induced obese mice without LC3-II detection by immunofluorescence [176]. Curiously, Nuñez et al., described diminished levels of phosphorylated mTOR together with increased Beclin-1 but also increased p62 levels in DIO mice compared to their lean counterparts [178]. Therefore, if results regarding LC3 and p62 are considered, evidence heads in the same direction (attenuated autophagy in high-fat diet-induced obesity). These discordant findings regarding LC3-II, beclin-1, or phosphorylated mTOR levels are remarkable. 

Another relevant issue is the precise step of the autophagic process which is affected during DIO. The study by Yoshizaki et al., suggested that autophagy is impaired from the early stages affecting autophagosome formation in DIO mice, as LC3-II (mainly degraded by lysosomes) did not accumulate after lysosomal inhibition [176]. By contrast, Mizunoe et al., reported an increased autophagosome formation induced by high-fat diet, but increased LC3-II and p62 (almost exclusively degraded by lysosomal pathway) after lysosomal inhibition. However, protease lysosomal dysregulation was seen, which suggests that only the latest step of autophagy might be attenuated in DIO animals [177]. These differences, similarly to other controversial studies, might be due to the different experimental approaches, highlighting the need for unifying criteria for the determination of the autophagic status [179]. Another issue could be differences in dietary intervention as the duration of the high-fat diet differed among the studies, suggesting a time-dependent adaptation in the autophagic response to nutritional stress [176,177,178]. It is also remarkable that there is a discordant interpretation by authors concerning the autophagic status in the different studies, e.g., increased levels of p62 are regarded as either attenuated [177] or enhanced autophagy [178], which makes it more difficult to draw clear conclusions. 

Regarding human studies, the general depicted trend is an increased WAT autophagy in obese and/or diabetic subjects (Table 1) [123,172,173,174,175,178,180,181]. It was independently found that higher mRNA and/or protein levels of several autophagic markers (Beclin-1, ATG5, ATG12, ATG7, LC3A and B, LC3-II, p62) and decreased mTOR expression in sWAT and/or vWAT from obese patients compared with lean individuals [123,172,173,174,175,178,180]. Notably, not all studies analyzed the same molecules and some of the markers, independently analyzed in the different studies, were not equally upregulated in every study or in each fat depot, which could be due to differences in study population characteristics. Taking into account the discordant findings regarding autophagy status in WAT from DIO mice and obese subjects, some authors aimed at confirming whether the autophagic flux was increased concomitantly to gene/protein expression of autophagy markers. Baseline accumulation of some autophagic proteins involved in cargo recognition and autophagosome formation, such as LC3 or p62 (almost exclusively degraded via lysosomes), could be indicative of two inverse situations: (1) increased autophagy flux or (2) impaired lysosomal degradation that leads to the accumulation of autophagosomes and associated proteins. Autophagic flux assays allowed to discern between these two situations [179]. To this end, in vitro experiments have been performed in the presence of lysosome inhibitors, finding that sWAT and vWAT explants from obese subjects and DIO mice had an enhanced autophagic flux and indicating concordant results with WAT gene or protein expression in obesity [173,177]. 

As WAT is composed of different cell types, it was raised whether the described alterations in total WAT autophagy from obese and diabetic individuals were due to adipocytes per se or to other WAT-resident cells such as macrophages (see Section 4.3). Then, several studies have analyzed autophagy in mature adipocytes vs. stromal vascular fraction (SVF) containing WAT resident cells other than mature adipocytes [172,173,180]. Although the different studies demonstrated the autophagy expression in mature adipocytes, it was not clear which fraction may contribute more to total WAT autophagy and differences between SVF and adipocytes were dependent on the autophagy-related gene measured. 

To be more precise, Kovsan et al., found higher ATG5, LC3A, and LC3B gene expression and LC3-II protein levels in visceral mature adipocytes than in SVF, although the donor phenotype is elusive [173]. Jansen et al., replicated the findings regarding ATG5 and also saw that ATG7 followed a similar trend in SVF and adipocytes from both sWAT and vWAT of healthy individuals. However, when analyzing other ATGs, i.e., ATG1 and ATG16L, an opposite trend towards lower expression in mature adipocytes than in SVF was found [172]. By contrast, Rodriguez et al., did not find any significant differences between visceral adipocytes and SVF from obese subjects in Beclin-1, ATG5, or ATG7. Despite not being statistically significant, ATG7 showed a trend towards higher expression in SVF than in adipocytes [180]. In view of these disparate results, no clear conclusion can be made regarding which WAT cellular fraction is more involved in total tissue autophagy or about the coordinated autophagic response of the different WAT cell populations to nutritional stress or metabolic signals in human obesity. 

Notably, none of the studies compared these two fractions according to the obesity degree or glycemic status. However, several studies did analyze autophagy in isolated adipocytes regarding obesity or the glycemic status [123,181]. Öst et al. found that subcutaneous adipocytes from diabetic obese patients compared to non-diabetic individuals (non-BMI matched) displayed attenuated mTOR expression concomitant to an increase in autophagic activity determined by autophagosome quantification, LC3 immunofluorescence and autophagic flux assay [123]. Conversely, Soussi et al. described the basal accumulation of p62 in obese subcutaneous adipocytes compared to lean adipocytes, but when assaying the autophagic flux, no changes in LC3 accumulation was seen, indicative of an already altered autophagic clearance in obesity [181]. The different group classification criteria in each study might be an explanation for discrepant results as the former prioritized the glycemic status whereas the latest, the obesity degree. This is indicative that glycemic status may be influencing WAT autophagy besides BMI. In fact, despite the established associations between the WAT expression of autophagic factors and obesity, it should be kept in mind that the discordant phenotypes regarding the obesity degree and the metabolic status (e.g., the metabolically healthy obese and metabolically unhealthy lean individuals) have yet to be studied. These phenotypes remark that the expansion capacity of WAT, rather than its size, would determine the development of metabolic alterations (see Section 2), and regulatory pathways involved in the WAT expandability are of relevance in determining metabolic health. As discussed throughout this review, autophagy is crucial in many of the processes required for healthy WAT expansion, so it is of great interest to gain insight into autophagic status according to the metabolic health of the individual. In this regard, few human studies have compared diabetic and/or insulin-resistant patients with normoglycemic subjects, finding an increased expression of autophagic factors in the former group [123,173,174,180]. Notably, only obese subjects with impaired glucose metabolism have been studied, hence it remains unknown whether metabolic alterations and more specifically glucose homeostasis, could per se drive dysregulated WAT autophagy. Some findings suggest that the glycemic status could have more weight than obesity as only T2D obese significantly differed from normoglycemic leans, normoglycemic obese and insulin-resistant obese participants, without significant differences between normoglycemic lean and normoglycemic obese subjects [180]. By contrast, others found a progressive augment of WAT autophagy from normoglycemic leans, non-diabetic obese, and T2D obese subjects [174]. Therefore, further in-depth studies with thorough patient phenotyping are needed to clarify this issue. In any case, it seems that altered autophagy in WAT is associated with a poorer metabolic profile as mRNA and/or protein levels of autophagic markers in sWAT or vWAT have been positively correlated with plasma triglycerides, cholesterol, free fatty acid, leptin levels and HOMA-IR, but negatively with adiponectin levels [172,173]. 

Given that the development of obesity-related comorbidities is more closely associated with central obesity (i.e., increased vWAT) than to peripheral obesity (i.e., increased sWAT), it is of interest to explore how autophagy is differentially altered in vWAT and sWAT during obesity. Kovsan et al., faced this issue and found that patients with intra-abdominal fat accumulation tended to have higher mRNA levels of ATG5, LC3A, and LC3B than patients with peripheral obesity. Accordingly, the authors reported augmented gene and protein expression of autophagic markers in vWAT vs. sWAT regardless of BMI or glycemic status [173]. Central obesity is typically related to hypertrophic and deleterious WAT expansion [90] as hypertrophied adipocytes have been associated with an impaired metabolic status (discussed in Section 2) [4,7,9,42]. In view of the association of dysregulated WAT autophagy with central obesity, several studies have analyzed the relationship between WAT autophagy and adipocyte cell size in obesity [173,175,181]. In agreement with the notion of increased autophagic flux under detrimental metabolic conditions, the expression of autophagic factors has been positively correlated with adipocyte size [173,181]. However, this was not confirmed by all the studies, with no significant correlation with adipocyte autophagic flux [175]. Methodological differences might account for these differences, and further studies should be performed to shed light on the relationship between autophagy and the promotion of adipocyte hypertrophy. 

Local and systemic low-grade inflammatory state is another hallmark of metabolic dysregulation [28,169,171,182]. It has been reported that autophagy inhibition in WAT or adipocytes triggers the inflammatory response (detailed in Section 4.3) [172,176]. These findings are challenging, taking into consideration that human obesity has been associated with higher autophagic expression in WAT. It has indeed been described that the enhanced expression of autophagic genes in sWAT and vWAT from obese subjects occurred in parallel with enhanced protein levels of proinflammatory cytokines and macrophage infiltration [174]. Increased LC3-II protein levels have been positively correlated with macrophage infiltration [172]. Notably, it remains unknown whether the enhanced LC3-II expression in these studies is due to enhanced autophagic flux or impaired lysosomal degradation. Some authors have hypothesized that the up-regulated autophagy in obesity (and/or insulin-resistant or diabetic states) is triggered as a protective mechanism against WAT inflammation [172], which would explain the putative controversial results. However, it should be also regarded that some models of ATG deficiency were protected from insulin resistance [78,80]. Another proposed hypothesis is that autophagy-independent pathways might be also modulating the inflammatory response [174]. 

The effect of weight-loss interventions on human WAT autophagy has also been studied, supporting the hypothesis about enhanced autophagy in obesity [178,183]. Nuñez et al., analyzed the expression of Beclin-1 in sWAT from obese patients before and 1 year after bariatric surgery, finding a significant reduction irrespective of the presence of diabetes. Beclin-1 expression levels after surgery were similar to those from non-diabetic lean subjects. Beclin-1 expression decrease was accompanied by a reduction in the autophagosome number [178]. Hence, it can be postulated that metabolic improvement after bariatric surgery comes together with sWAT autophagy attenuation. Conversely, Soussi et al., indicated an impaired autophagic flux in adipocytes from obese patients which recovers after bariatric surgery [181]. However, it is worth mentioning that the authors compared LC3-II levels upon lysosomal inhibition between pre- and postsurgical conditions, but it was not specified whether autophagic flux was modified in each condition due to lysosomal inhibition. Then, it is not clear whether autophagic flux was or was not impaired in each condition. Modulation of sWAT autophagy by dietary intervention has also been reported in obese patients. Evidence suggests that this modulation depends on the type of dietary fat consumed. Specifically, an enriched monounsaturated fatty acid diet led to an increase in sWAT Beclin-1 and ATG7 gene expression [183]. This increase in gene expression of autophagic markers was concomitant with a decrease in postprandial oxidative stress in sWAT [184], so the protective role of autophagy was again claimed, in this case, against nutritional stress. The authors hypothesized that monounsaturated fatty acids elicit a better adaptive response than saturated fatty acids or carbohydrates [183]. Unfortunately, changes in the expression of autophagic genes were not analyzed regarding diet-induced metabolic changes, with the systemic effect of diet-induced autophagic sWAT response remaining unknown. 

## 6. Concluding Remarks

Experimental data have revealed the involvement of autophagy in the different processes triggered during WAT expansion. Autophagy plays a relevant role in white adipogenesis, likely favoring hyperplasic expansion of WAT. In addition, the protective role of autophagy in the control of the inflammatory response has also been demonstrated in WAT and adipocytes. There is evidence suggesting that autophagy may be involved in ECM remodeling, though a wider overview and more in-depth studies are required to gain insight into the contribution of autophagy in WAT fibrosis. Given this general outline, autophagy activation in WAT may be beneficial for tissue homeostasis and healthy expansion. However, it should be kept in mind that not all studies could confirm the sense in which autophagy participates in each of these processes. Several issues may be influencing the discordant findings. Firstly, many of the contradictory results come from animal models of genetic autophagy deletion which target disparate autophagy-related genes. Thus, it should be considered that different autophagic molecules might have different weight on the global autophagic process, and compensatory mechanisms should not be discarded. Furthermore, non-canonical functions for autophagic proteins have been also reported [102] and then, the consequences of single autophagic gene deletion might also be the result of their autophagy-independent roles. Many regulatory pathways other than autophagy control WAT physiology and the participation of autophagy should be regarded as a part of the whole WAT regulatory network. 

Once the implication of autophagy in WAT expandability has been established, the next question is whether the maladaptive WAT expansion in obesity and/or metabolic disruption is related to WAT autophagy alterations. In this regard, human studies have given some clues. Most of the studies point to an overactivation of WAT autophagy in obesity. When put into the context of experimental data, several assumptions can be raised. Autophagy overactivation may result in hyperplasic WAT expansion because of enhanced white adipogenesis. This may be somewhat contradictory if considered WAT hyperplasia as the healthy way to expand. Then, autophagy can be regarded as a protective mechanism to avoid WAT maladaptation to nutritional stress. This would also explain enhanced autophagy despite the increased inflammation in dysfunctional WAT (that it might be even worse in the case of decreased autophagy). On the other hand, it should be borne in mind that metabolic dysregulation is not always associated with obesity. Current studies have only addressed the case of obese patients with altered glycemic status. The few studies that included non-diabetic obese patients showed that autophagy overactivation might be not necessarily linked to obesity per se. Taking into consideration that autophagy is highly regulated by nutritional sensors, particularly by insulin, the insulin-resistant state or diabetes are likely influencing WAT autophagy even more than obesity itself. However, this question remains unsolved and the autophagic status should also be assessed in non-obese diabetic patients to elucidate the actual relationship between autophagy, glycemic dysregulation, and obesity. 

Given the implication of autophagy in WAT and other metabolic tissues, it has been proposed as a putative therapeutic target. However, many gaps remain to be filled before targeting autophagy to treat metabolic diseases. For instance, the inverse trend in autophagy activation or suppression in the different metabolic organs related to obesity or diabetes. This highlights the necessity of tissue-targeted strategies to manage autophagy. But even in the same tissue, cell heterogeneity also makes this approach difficult, as autophagy has different functions depending on cell type. This takes special relevance in WAT where fibroblasts, preadipocytes, mature adipocytes, and immune cells coexist. Further comprehensive studies should be developed to shed light on the tangled network which links autophagy with each regulatory pathway in WAT. 

## Figures and Tables

**Table 1:** Human studies analyzing autophagy state in obesity and/or diabetes.

Study [Reference]	Design	WAT Depot	Tissue/Cells/Explant	Methodological Approach	Autophagic Markers	Main Results
Öst et al. [123]	Study groups: (1) T2D and BMI > 27 kg/m^2^ (n = 7) vs. (2) Non-T2D (non-BMI matched) (n = 7)	Subcutaneous	- Isolated adipocytes	- TEM - Immunofluorescence - Immunoblotting - Real-time qPCR	LC3 mTORC Autophagosome	T2D led to: - ↑ autophagosome number (TEM). - ↑ autophagic flux (accumulation of LC3). - ↓ lipofuscin particles. - = mTOR protein expression.
Kovsan et al. [173]	Cohort 1: Non-obese (BMI < 30; n = 15) vs. Obese (BMI > 30; n = 50) Cohort 2: Lean (BMI < 25; n = 66); Subcutaneous obesity (BMI > 30; n = 88) vs. Visceral obesity (BMI > 30; n = 42). Cohort 3: IS obese (BMI > 40; n = 30) vs. IR obese (BMI > 40; n = 30)	-Subcutaneous -Visceral	- Total tissue - Explant (n = 1 Ow) - SVF and adipocytes (only vWAT) (n = 24).	- Real-time qPCR - Immunoblotting - Immunofluorescence (explant). - Autophagic flux analysis	ATG5 ATG12 LC3A LC3B LC3-I & II p62 NBR1	Total WAT: - ↑ ATG5 & LC3- II protein expression in vWAT vs. sWAT in obesity. - ↑ ATG12-ATG5 complex protein expression in vWAT vs. sWAT, regardless of BMI. - ↑ ATG5 and LC3B mRNA levels in vWAT vs. sWAT. - ↑ mRNA ATG5, LC3A and LC3B levels in obese vs. lean. - ↑ mRNA ATG5, LC3A and LC3B levels in IR obese vs. IS obese. mRNA/protein levels of ATG5, LC3B/LC3-II higher in adipocytes vs. SVF. Explant: - ↑ LC3-positive dots vWAT vs. sWAT. - ↑ autophagic flux in obesity (p62 accumulation).
Jansen et al. [172]	Study groups: (1) Obese (BMI = 27–35; n = 16) (2) Lean (BMI = 20–25; n = 17)	- Subcutaneous - Visceral	- Total tissue - Explant	- Immunoblotting	LC3-II	Total sWAT - ↑ LC3-II in obesity. - Positive correlation between LC3-II levels and BMI, HOMA-IR, macrophage infiltration. Explant (vWAT & sWAT): - Autophagy inhibition increased proinflammatory response.
Rodríguez, A. et al. [180]	Study groups: (1) NG Lean (BMI < 25; n = 55) (2) NG Obese (n = 66)* (3) IGT Obese (n = 37) * (4) T2D Obese (n = 36)* * Obese: BMI ≥ 30	- Visceral	- Total tissue - Adipocytes - SVF	- Real-time qPCR	BECN1 ATG5 ATG7	Total vWAT: ↑ BECN1 and ATG7 mRNA levels in T2D Obese vs. NG lean and obese. Adipocytes vs. SVF: No significant differences. Adipocytes: - ↑ BECN1, ATG5 and ATG7 mRNA levels upon TNFα stimulus. - ↓ ATGs mRNA upon acylated ghrelin stimulus.
Nuñez, C.E et al. [178]	Study groups: (1) Non-T2D obese(BMI = 43 ± 4.3; n = 9)* (2) T2D (BMI = 32 ± 2.2; n = 6)* (3) Lean control (BMI = 23 ± 2.7; n = 8) * Prospective follow-up ~1 year after surgery	Subcutaneous	- Total tissue - Explant	- Immunoblotting - - TEM	Beclin Autophagosome	- ↑ Beclin in obese groups vs. lean group. - ↓ Beclin after surgery (non-differences between T2D and non-T2D). - ↓ autophagosome after surgery.
Kosacka, J. et al. [174]	Study groups: (1) Lean (BMI < 25; n = 20). (2) Non-T2D Obese (BMI > 30; n = 20). (3) T2D Obese (BMI > 30; n = 20)	- Subcutaneous - Visceral	- Total tissue	- Immunoblotting - Immunofluorescence - Real-time qPCR - TEM	LC3-I & II ATG5/12 p62 mTOR (mRNA: LC3A & B, ATG5) Autophagosome	General trends: - gradual increase in LC3, ATGs & p62 from group (1) to (3) in vWAT and sWAT. - Parallel increase in inflammatory and caspase-dependent apoptotic markers. - Higher number of autophagosomes in obese vs. lean.
Soussi, H. et al. [181]	Study groups: (1) Lean (BMI = 20–23; n = 12). (2) Obese (BMI = 34–79; n = 24). * Subcohort from group 2: Obese for bariatric surgery follow up (3–12 months) (n = 9).	- Subcutaneous	-Isolated adipocytes	- Real-time qPCR. - Immunoblotting. - Autophagic flux.	LC3-I & II p62	- ↑ p62 mRNA and protein levels in obesity. - ↓ absolute LC3-II levels in obese vs. lean after lysosomal inhibition. - ↑ absolute LC3-II levels post-surgery vs. pre-surgery after lysosomal inhibition.
Xu, Q. et al. [175]	Study groups: (1) Ow/Obese patients (BMI > 25–41.7; n = 17) with or without IGT. (2) NG lean (BMI ≤ 25; n = 9).	- Subcutaneous	- Total tissue. - Differentiated adipocytes from hMADS (non-clinical phenotype indicated).	- Real-time PCR - Autophagic flux	mRNA: ULK1-2 BECN1 ATG 5, 7 & 12 LC3A-B Protein: LC3-I & II p62	Total sWAT: ↑ ATGs mRNA levels in Ow/obese group. Adipocytes: ↑ LC3-II and = p62 protein levels after lysosomal and lipolysis inhibition vs. non-lipolysis inhibition.

BMI, Body Mass Index; hMADS, human multipotent adipose-derived stem cells; IGT, impaired glucose tolerance; IR, Insulin resistant; IS, Insulin Sensitive; NG, normoglycemic; Ow, Overweight; SVF, stroma vascular fraction; sWAT, subcutaneous white adipose tissue; T2D, Type 2 Diabetes; TEM, transmission electron microscopy; vWAT, visceral white adipose tissue; WAT, White Adipose Tissue. BMI units: Kg/m^2^.
